# Multifunctional liposomes Co-encapsulating epigallocatechin-3-gallate (EGCG) and miRNA for atherosclerosis lesion elimination†

**DOI:** 10.1039/d3na00369h

**Published:** 2023-10-30

**Authors:** Dandan Li, Danni Liu, Yaoqi Wang, Qi Sun, Ran Sun, Jie Zhang, Xiaoxuan Hong, Ran Huo, Shuang Zhang, Chunying Cui

**Affiliations:** a Department of Pharmaceutics, School of Pharmaceutical Science, Capital Medical University No. 10 Youanmenwai Street, Fengtai Beijing 100069 People's Republic of China ccy@ccmu.edu.cn zshuang@ccmu.edu.cn +86-10-8391-1673 +86-10-8391-1668; b Engineering Research Center of Endogenous Prophylactic of Ministry of Education of China Beijing 10069 People's Republic of China; c Beijing Area Major Laboratory of Peptide and Small Molecular Drugs Beijing 10069 People's Republic of China

## Abstract

Atherosclerosis (AS) is a chronic inflammatory disease, characterized by a lipid accumulated plaque. Anti-oxidative and anti-inflammation and lipid metabolism promoting therapeutic strategies have been applied for atherosclerosis treatment. However, the therapeutic effect of a single therapeutic method is limited. It is suggested that a combination of these two strategies could help prevent lipid accumulation caused by inflammation and oxidative stress, and also promote lipid efflux from atherosclerotic plaque, to normalize arteries to the maximum extent. Hence, a strategy involving a multifunctional liposome co-encapsulating an antioxidant and anti-inflammatory drug epigallocatechin-3-gallate (EGCG) and a lipid-efflux-promoting gene miR-223 was established. The system (lip@EGCG/miR-223) could encapsulate miR-223 in core areas of the liposomes to provide a protective effect for gene drugs. Moreover, lip@EGCG/miR-223 was smaller in size (91.28 ± 2.28 nm characterized by DLS), making it easier to target AS lesions, which have smaller vascular endothelial spaces. After being efficiently internalized into the cells, lip@EGCG/miR-223 exhibited excellent antioxidant and anti-inflammatory effects *in vitro* by eliminating overproduced ROS and decreasing the level of inflammatory cytokines (TNF-α, IL-1β, and MCP-1), which was due to the effect of EGCG. Besides, the lipid-efflux-promoting protein ABCA1 was upregulated when treated with lip@EGCG/miR-223. Through the two therapies mentioned, lip@EGCG/miR-223 could effectively inhibit the formation of foam cells, which are a main component of atherosclerotic plaques. In AS model mice, after intravenous (*i.v.*) administration, lip@EGCG/miR-223 was effectively accumulated in atherosclerotic plaques, and the distribution of drugs in the heart and aorta compared to that in the kidney was significantly increased when compared with free drugs (the ratio was 6.27% for the free miR-223-treated group, which increased to 66.10% for the lip@EGCG/miR-223-treated group). By decreasing the inflammation level and lipid accumulation, the arterial vessels in AS were normalized, with less macrophages and micro-angiogenesis, when treated with lip@EGCG/miR-223. Overall, this study demonstrated that lip@EGCG/miR-223 could be developed as a potential system for atherosclerosis treatment by a combined treatment of antioxidant, anti-inflammatory, and lipid-efflux-promoting effects, which provides a novel strategy for the safe and efficient management of atherosclerosis.

## Abstract 2

A liposome co-loaded with an antioxidant and anti-inflammatory drug EGCG and a lipid-efflux-promoting gene miR-223 is constructed to eliminate AS plaques effectively, and provide a novel strategy for the safe and efficient management of AS.

## Graphical abstract

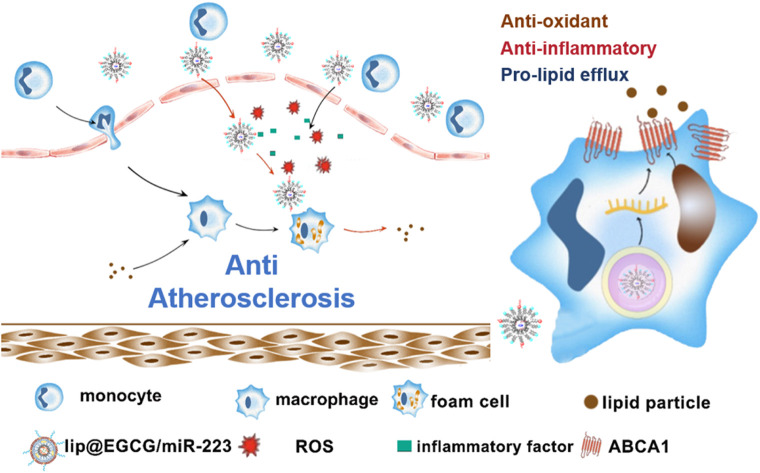

## 1. Introduction

Cardiovascular disease (CVD) is the leading cause of mortality worldwide.^1^ Atherosclerosis (AS) is a leading cause of myocardial infarction, stroke, peripheral artery disease, and other CVDs, which is characterized by the accumulation of lipids, cells, and the extracellular matrix in arterial intima.^2^ Since AS is a disease caused by lipid disorder, the current clinical treatments against AS mostly focus on preventing the growth of atherosclerotic plaque by lowering the levels of low-density lipoprotein (LDL) and cholesterol.^3^ However, this treatment cannot eliminate the lipid plaques that have already formed. With the in-depth understanding of the pathogenesis of AS, increasing number of studies have shown that inflammation is closely related to the initiation and the progression of AS.^4–6^ In the early stage of AS, macrophages phagocytose large amounts of ox-LDL, leading to foam cells that accumulate as plaques in the arterial wall.^7–9^ On the other hand, excess ROS production is the leading cause and a key feature of AS.^10,11^ As reported, ROS will induce LDLs to oxidize and form ox-LDLs, which exhibit proinflammatory and toxic properties, and aggravate endothelial damage.^12^ Therefore, a promising strategy for the management of AS and other CVDs is the suppression of the inflammation and oxidation environment followed by the elimination of the lipids in foam cells at the same time.

The natural compound epigallocatechin-3-gallate (EGCG) is the major catechin in green tea and accounts for 50–80% of the total catechins.^13^ EGCG has multiple phenolic hydroxyl groups that can be easily oxidized into phenols, endowing it with a high antioxidant activity and free-radical-scavenging capacity.^14^ EGCG also possesses high anti-inflammatory efficacy by inhibiting the secretion of proinflammatory cytokines (TNF-α, IL-1β, and MCP-1).^15^ Despite its advantages, EGCG has still found limited application as an antioxidant and anti-inflammatory *in vivo*. The reasons for this are first, EGCG is not stable and can be easily oxidized at high temperature and in neutral or alkaline solutions;^16^ second, the bioavailability of EGCG is low, with its poor absorption, rapid metabolism, and fast elimination *in vivo*.^17^ Therefore, it is critical to design an effective strategy to increase the stability and bioavailability of EGCG for exertion of its biological activities efficiently.

Gene therapy has shown prospects in the treatment of various diseases, such as tumors, and cardiovascular diseases.^18,19^ Among the genes, miR-223 is a microRNA involved in the progression of AS, and treatment with miR-223 could inhibit the formation of foam cells by increasing the level of the ATP-binding cassette transporter A1 (ABCA1), whose main function is to efflux cholesterol by the reverse cholesterol transport pathway (RCT), thus reducing the progress of AS.^20^ Due to its large size and negative charge, it is difficult to introduce naked miR-223 into cells and produce therapeutic effects at specific sites. Therefore, developing safe and efficient gene-delivery systems *in vivo* has become a key challenge for gene therapy in clinic.

Nano-drug delivery systems have been applied for AS treatment because of the permeability and retention (EPR) effect developed during the formation of atherosclerotic lesions.^21,22^ Liposomes are a widely applied nano-drug delivery system, which have the advantages of high biocompatibility, excellent drug-loading capacity, and controllable size.^23,24^ Moreover, as nanoparticles composed of lipid molecules, they have a natural ability to spontaneously accumulate in AS, making them an ideal drug-delivery system for AS treatment.^25^ Liposomes are also an attractive platform for creating gene-delivery systems. However, most liposomes applied for gene delivery are cationic particles, whereby genes are absorbed on the surface of the liposomes. So the exposed cationic membranes could induce aggregation, increase cellular and systemic toxicity, as well as fall short of offering effective protection for genes.^26^

In this study, a novel lipid nano-drug-delivery system was prepared by an improved thin film hydration method, which involved co-encapsulation with EGCG and miR-223. It was intended that, by the dual effects of antioxidant and anti-inflammatory as well as lipid metabolism promotion, the nano-drug delivery system could eliminate atherosclerotic lesions efficiently. Our experimental results proved that lip@EGCG/miR-223 could significantly alleviate the accumulation of lipid plaques at the site of atherosclerotic lesions and restore diseased blood vessels into a normal state.

## 2. Materials and methods

### 2.1. Materials

1,2-Dioleoyl-3-trimethylammonium propane (DOTAP) was purchased from Corden Pharma Switzerland LLC (Switzerland). Phosphatidylinositol (PI), distearoyl phosphoethanolamine-PEG2000 (DSPE-PEG 2000), cholesterol, and Tween-80 were purchased from Shanghai Macklin Biochemical Co., Ltd (Shanghai, China). Epigallocatechin gallate (EGCG) was purchased from Send Pharm (Shanghai, China).

The RAW 264.7 cell line was obtained from the Cell Bank of Chinese Academy of Medical Sciences. miR-223, Cy5-miR-223, and the negative control miR-223 (NC) were purchased from GenePharma Co., Ltd (Shanghai, China). DMEM medium and trypsin were purchased from HyClone Laboratories Inc (Logan, UT, USA). Fetal bovine serum (FBS) was purchased from Gibco (Australia). Penicillin and streptomycin were obtained from Sigma-Aldrich (St Louis, MO, USA). Lipopolysaccharide (LPS) and oxidized low-density lipoprotein (ox-LDL) were purchased from Shanghai Yuanye Biotechnology Co., Ltd (Shanghai, China). DPPH radical and Oil red O were purchased from Shanghai Aladdin Reagent Co., Ltd (Shanghai, China). Dichlorofluorescin diacetate (DCFH-DA) was obtained from MedChemExpress (New Jersey, USA). LysoTracker Green and Hoechst 33 342 were purchased from Invitrogen (USA).

The sense chain sequence of miR-223 was UGUCAGUUUGUCAAAUACCCCA; while the anti-sense chain sequence of miR-223 was GGGUAUUUGACAAACUGACAU.

All other chemicals and reagents were of analytical grade.

### 2.2. Agarose gel retardation assay

The agarose gel retardation assay was applied to analyze the miR-223 binding affinity of liposome. DOTAP-miR-223 was prepared at different mass ratios (1 : 1.5, 1 : 1.25, 1 : 1, 1 : 0.75, 1 : 0.5). First, 20 μL of the liquid supernatant was drawn, which was collected above to load into agarose gel wells (containing 1% agarose gel with 2 mg mL^−1^ EtBr) in TBE buffer. After electrophoresis under 120 V for 30 min, the results were measured by a gel imaging system (ChemiDoc, Bio-Rad, USA).

### 2.3. Synthesis of lip@EGCG/miR-223

Liposome was prepared by an improved thin film hydration method. First, 0.033 mg (1OD) of miR-223 was dissolved in 200 μL deionized water. Next, 0.044 mg DOTAP (DOTAP : miR-223 = 1 : 0.75, mass ratio) was dissolved in 200 μL chloroform, and 400 μL methanol was added, and the mixture was gently mixed to form a monophase. After 30 min incubation at room temperature, 200 μL deionized water and 200 μL chloroform were added to form two separate phases. Upon centrifugation at 800*g* for 8 min at 5 °C, the organic phase containing the DOTAP-miR-223 complex was extracted. PI (0.117 mg), DSPE-PEG2k (0.167 mg), chol (0.217 mg), and Tween-80 (0.167 mg) were added into the extracted organic phase. Then, 1 mL chloroform was added, and the organic phase was evaporated at 40 °C in a rotary evaporator for 20 min to form a film on the wall of the bottle. Next, EGCG solution (2 mg EGCG in 1 mL deionized water) was added into the bottle. The mixture was kept under vacuum condition in the rotary evaporator for another 30 min. The obtained solution was ultrasonicated in a water bath for 5 min, probe ultrasonicated for 2 min (power: 25%, stop for 3 s after 3 s intervals) and extruded through a 100 nm polycarbonate membrane 11 times to prepare lip@EGCG/miR-223.

### 2.4. Characterization of lip@EGCG/miR-223

The surface morphology of lip@EGCG/miR-223 was examined by transmission electron microscopy (TEM, JEM-2100, JEOL, Japan). After the liposome was dispersed in deionized water, the hydrodynamic diameter, PDI, and zeta potential of the liposome were measured by dynamic light scattering (DLS, Zetasizer-Nano-ZS90, Malvern, UK).

### 2.5. Evaluation of the miR-223 and EGCG encapsulation efficiency

The miR-223 and EGCG encapsulation efficiency (EE) of the liposomes was determined by ultrafiltrating the liposomes entrapping Cy5-miR-223 and EGCG with DEPC water. The encapsulated Cy5-miR-223 and EGCG were collected by ultrafiltration, and quantified by constructing a standard curve. The fluorescence intensity of Cy5-miR-223 was measured by a fluorescence spectrophotometer (ex 650 nm, em 670 nm, RF-6000, SHIMADZU, Japan). The absorbance of EGCG was measured by a UV spectrophotometer (UV-2600, SHIMADZU, Japan) at a wavelength of 276 nm.

The miR-223 and EGCG EEs were calculated using the following formula:
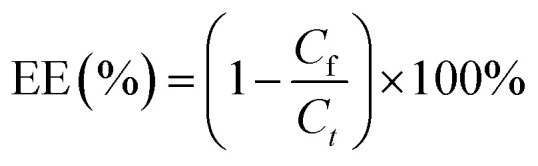
where *C*_f_ and *C*_*t*_ denote the mass of unencapsulated drug and total drug in the liposome, respectively.

### 2.6. Anti-RNase a degradation assay

Naked miR-223 (1 μg) and lip@EGCG/miR-223 (loaded with 1 μg miR-223) were added into RNase A solution (10 μg mL^−1^) separately at 37 °C for 30 min. Then, 5 μL EDTA solution (5 mM) was applied to terminate RNase A degradation at a set point time. Finally, 20 μL heparin sodium (0.8 mg mL^−1^) was added at 37 °C for 30 min to replace the miR-223 in the lip@EGCG/miR-223. The samples at different time points were manipulated according to the agarose gel retardation assay mentioned above.

### 2.7. DPPH scavenging capability

To evaluate the free-radical-eliminating capability, lip@EGCG/miR-223 with different concentrations of EGCG (0, 10, 20, 50, 100, and 200 μg mL^−1^) was dispersed in 2 mL of methanol, and 1 mL DPPH˙ (100 μg mL^−1^) was added and incubated for 30 min at room temperature in the dark. Subsequently, the concentration of the residual free radicals was determined using a UV spectrophotometer at 517 nm, and the scavenging rate (*I*) was calculated as follows:
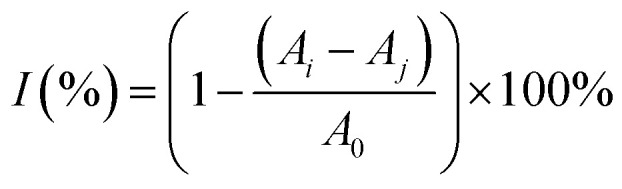
where *A*_0_ is the absorbance of DPPH˙ when the solution to be measured was not added, *A*_*j*_ is the absorbance of the solution to be measured, and *A*_*i*_ is the absorbance of DPPH˙ after adding the solution to be measured.

### 2.8. Cell culture

Raw264.7 cells were cultured in DMEM medium containing 10% FBS, and 1% penicillin and streptomycin, and kept at 37 °C with 5% CO_2_. The cells were subcultured 2–3 times a week till they reached 80% confluence.

### 2.9. Cellular uptake and lysosomal escape

To evaluate cellular uptake and lysosomal escape, Cy5-labeled miR-223 was applied. First, Raw 264.7 cells (2 × 10^5^ cells) were planted in a confocal dish and incubated for 12 h. The cells were then treated with free Cy5-miR-223 (160 nM) and lip@EGCG/Cy5-miR-223 (contain 160 nM miR-223) separately for 2, 4, and 6 h. The medium was discarded and the cells were washed with PBS three times. Then the cells were fixed with 4% paraformaldehyde for 10 min and stained with LysoTracker Green and Hoechst 33 342 to observe the cell nuclei and lysosomes, respectively. Finally, the cells were observed by confocal laser scanning microscopy (CLSM, TCSSP5, Leica, Wetzlar, Germany).

### 2.10. ROS levels in macrophages

RAW 264.7 cells at a density of 2 × 10^5^ cells per well were seeded in a 12-well plate and incubated for 12 h. After the cells were treated with different concentrations of lip@EGCG/miR-223 containing 10, 50, and 100 μg mL^−1^ EGCG for 6 h, the cells were stimulated with LPS (20 μg mL^−1^) for 8 h. The cells treated with medium were considered as the negative control group, and the cells without treatment with lip@EGCG/miR-223 were considered as the positive control group. Then the medium was discarded and the cells were washed three times with PBS. Subsequently, DCFH-DA (50 μM) diluted with serum-free DMEM was added and incubated for 30 min. After washing with PBS three times, the cells were infiltrated with PBS and observed by fluorescence microscopy (Primovert, Zeiss, Germany).

### 2.11. Investigation of anti-inflammatory effect *in vitro*

RAW 264.7 cells were seeded in a 24-well plate at the density of 1 × 10^5^ cells per well and incubated for 12 h. The negative control group was treated with fresh medium, and the other groups were stimulated with LPS (20 μg mL^−1^) for 8 h. Then the cells were treated with free EGCG (100 μg mL^−1^), lip@EGCG (containing 100 μg mL^−1^ EGCG), or lip@EGCG/miR-223 (containing 100 μg mL^−1^ EGCG) for 6 h, and the cells without treatment were considered as the positive control group. The supernatant of each experimental group was collected, and the typical inflammatory cytokines, including tumor necrosis factor-α (TNF-α), interleukin-1β (IL-1β), and monocyte chemoattractant protein-1 (MCP-1), were determined by an ELISA kit (Beyotime, Shanghai, China), while the levels of total protein were quantified by a BCA protein assay kit (Beyotime, Shanghai, China).

### 2.12. Promoting foam cell lipid efflux *in vitro*

RAW 264.7 cells at a density of 2 × 10^5^ cells per well were seeded in a 12-well plate and incubated for 12 h as above. The negative control group was treated with fresh medium, and the other groups were stimulated with ox-LDL (25 μg mL^−1^) for 48 h. Then, the cells were treated with free miR-223 (10 μg mL^−1^), lip@miR-223 (containing 10 μg mL^−1^ miR-223), or lip@EGCG/miR-223 (containing 10 μg mL^−1^ miR-223) for 8 h, and the cells without treatment were considered as the positive control group. After the medium was discarded, the cells were rinsed with PBS three times, fixed with 4% paraformaldehyde for 30 min at 4 °C, and washed with PBS three times again. Subsequently, the cells were stained with 0.3% ORO in 60% isopropanol for 30 min, and observed by fluorescence microscopy. In addition, intracellular ORO was extracted by isopropanol and the ORO concentration was determined by measuring the absorbance of the solutions at 492 nm *via* UV-visible spectrometry.

### 2.13. Expression of ABCA1 mRNA tested by RT-qPCR

Investigation of miR-223 expression was performed by RT-qPCR. RAW 264.7 cells were seeded in a 12-well plate at a density of 2 × 10^5^ cells per well and incubated for 12 h. The cells were treated with free miR-223, lip@miR-223 or lip@EGCG/miR-223 with different concentrations of miR-223 (2 μg mL^−1^) for 6 h. The negative control group was treated with fresh medium. Then, the medicated medium was discarded and replaced with complete fresh medium of equal volume, and the cells were further incubated for 42 h. Subsequently, Trizol reagent was added to extract the total RNA from the cells, and the RNA concentration was examined by a Nano Drop 1000 system (Thermo Scientific, USA). Also, 2 μg RNA was reverse transcribed into cDNA in the GeneAmp® PCR System 9700 (Applied Biosystems, USA) and the cDNA concentration was examined by the Nano Drop 1000 system again. Finally, RT-qPCR was performed to amplify the cDNA in the Real Time PCR System 7500 (Applied Biosystems, USA), and the mRNA expressions levels were compared with the housekeeping gene GAPDH as the internal control. The relative quantity of mRNA was calculated with the average threshold cycle (*C*_*t*_) by the delta–delta *C*_*t*_ (2^−ΔΔ*C*_*t*_^) method.

### 2.14. Expression of ABCA1 protein tested by ELISA

Investigation of miR-223 expression was performed by ELISA. RAW 264.7 cells were seeded in a 24-well plate at a density of 1 × 10^5^ cells per well and incubated for 12 h. The cells were treated with free miR-223, lip@miR-223, or lip@EGCG/miR-223 with different concentrations of miR-223 (2 μg mL^−1^) for 6 h. The negative control group was treated with fresh medium. Then the medicated medium was discarded and replaced with complete fresh medium of equal volume, and the cells were further incubated for 42 h. After washing with PBS, the cells were obtained and extracted by RIPA solution. The concentration of total protein was measured using a BCA protein assay kit, and the amount of ABCA1 protein was measured using a Mouse ABCA1 ELISA kit.

### 2.15. Animals

All animal care and experiments were conducted in line with the Guide for the Care and Use of Laboratory Animals proposed by the National Institutes of Health. All procedures and protocols were approved by the (Institutional Animal Ethics Committee of Capital Medical University). Apolipoprotein E-deficient (ApoE^−/−^) mice (about 8 weeks old, 20 g) were purchased from the Animal Department of Capital Medical University (Beijing Laboratory Animal Center, Beijing, China).

First, the atherosclerosis (AS) mice model was established by feeding the mice a high fat diet (HFD) containing 20% fat, 20% sugar, and 1.25% cholesterol for 8 weeks. Then, the mice were divided into three groups and treated with 5% glucose solution, and a mixture solution of miR-223 and EGCG and lip@EGCG/miR-223 by intravenous injection (the concentration of miR-223 was 1 mg kg^−1^, and the concentration of EGCG was 10 mg kg^−1^). The frequency of drug administration was once every four days, for a total of five doses. After three weeks of treatment, all the mice were sacrificed for the subsequent experiments.

### 2.16. Targeting effect of lip@EGCG/miR-223 *in vivo*

To test the targeting effect of lip@EGCG/miR-223 in the AS mice model, Cy5-labeled miR-223 was applied for animal fluorescence imaging. The model mice were treated by intravenous injection with free Cy5-miR-223 or lip@EGCG/Cy5-miR-223 for each mouse. After 8 h, the mice were perfused transcardially with 30 mL PBS and 30 mL 4% paraformaldehyde under anesthesia, and the aorta and the primary organs, including the heart, liver, spleen, lung, and kidney, were isolated and imaged by a fluorescence imaging system (IVIS Spectrum, Waltham, MA, USA). Finally, the fluorescence results were quantified by Image J software.

### 2.17. Investigation of the lipid-efflux promotion *in vivo*

After the AS model mice were euthanized, the aorta was excised and the surrounding adipose tissue was removed. Subsequently, the aorta was fixed with 4% paraformaldehyde overnight. After that, the aorta, which was first washed with PBS, was cut longitudinally and stained with 0.3% ORO for 1 h to quantify the plaque area. Then the aorta was made into 5 μm frozen sections by freezing microtome, and stained with ORO to observe the distribution of lipids in the aorta.

### 2.18. Histology and immunohistochemistry

After fixing in 4% paraformaldehyde overnight, aortic arch sections (5 μm thickness) in paraffin were prepared. Antibodies to IL-1β and TNF-α were incubated with the sections to investigate the anti-inflammatory effect of lip@EGEG/miR-223, and antibodies to CD68 were applied to explore the distribution of macrophages in the aortic arch areas, and antibodies to CD31 were applied to observe the morphology of the aortic vessels. All of the sections were stained with hematoxylin to observe the structures of the tissues. Images of the stained sections were observed by a fluorescence microscope (Nikon Eclipse Ti-SR, Japan), and the semiquantitative analysis of the histological images was performed with the ImageJ software.

### 2.19. H&E staining

After the mice were euthanized, the main organics, including the heart, liver, spleen, lung, and kidney, were collected and made into sections in paraffin. Then the sections were stained with Hematoxylin–Eosin (H&E) and observed to investigate the organic toxicity of lip@EGCG/miR-223 by fluorescence microscopy.

### 2.20. Statistics

The data were expressed as the mean ± SD (standard deviation). The *t*-test was used for statistical analysis. A value of *p* < 0.05 was considered statistically significant, and a value of *p* < 0.01 was considered very significant.

## 3. Results and discussion

### 3.1. Synthesis and characterization of lip@EGCG/miR-223

**Fig. 1:**
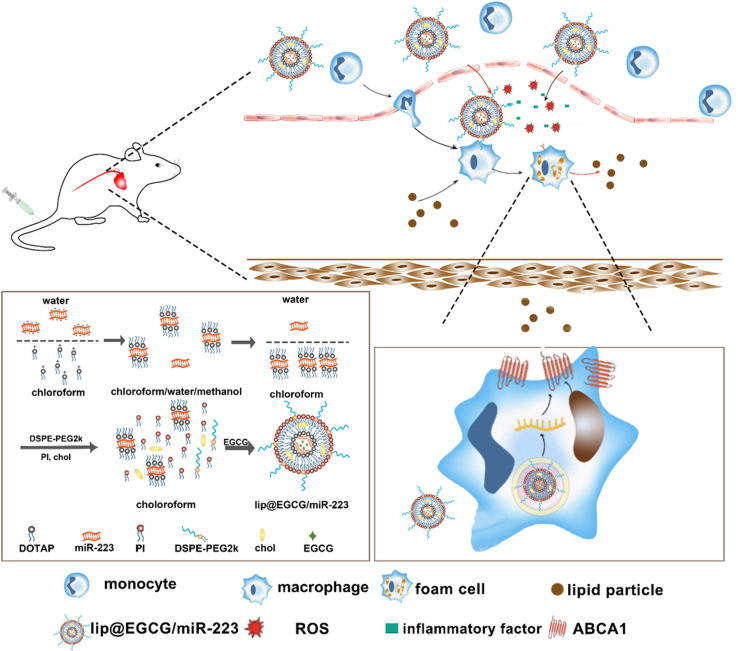
Schematic illustration of the mechanisms and preparation of lip@EGCG/miR-223, in which lip@EGCG/miR-223 was prepared by a thin film hydration method, and could be effectively delivered to the AS lesion site by an EPR effect. EGCG in the lip@EGCG/miR-223 can then reduce the level of ROS and the expression of inflammatory factors to exert antioxidant and anti-inflammatory effects. At the same time, lip@EGCG/miR-223 absorbed by macrophages can promote lipid efflux and eliminate lipid plaques through miR-223 expression.

**Fig. 2:**
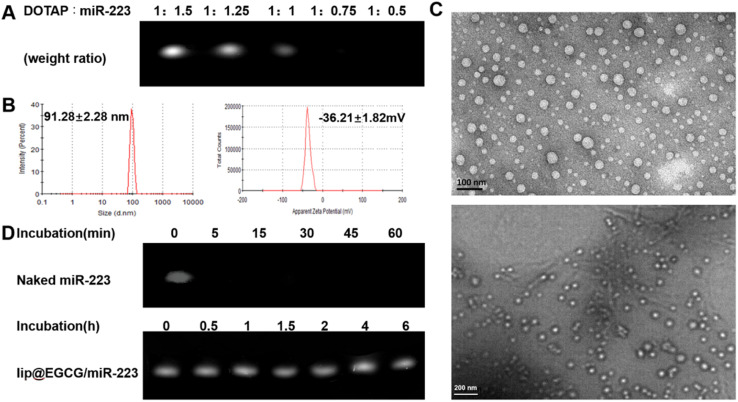
Characterization of the physical and chemical properties of lip@EGCG/miR-223. (A) Agarose gel retardation assays of miR-223 complexed with DOTAP. (B) Hydrodynamic diameter and the zeta potential of lip@EGCG/miR-223. (C) TEM images of lip@EGCG/miR-223 at different magnifications. (D) Degradation of naked miR-223 and lip@EGCG/miR-223 against RNase A.

The preparation process of lip@EGCG/miR-223 is shown in Fig. 1. First, the positively charged cationic lipid DOTAP and the negatively charged miR-223 were bound together by electrostatic interaction, and DOTAP was used to extract miR-223 from the aqueous to the organic phase by the method described in Bligh and Dyer in the monophase.^27^ The binding capacity of DOTAP and miR-223 was determined by agarose gel retardation assay. As shown in Fig. 2A, the intensity of the miR-223 band gradually decreased when the mass ratio of DOTAP to miR-223 increased. The band totally disappeared when the ratio was 1 : 0.75, which signified that miR-223 was completely combined with DOTAP. According to the results, we determined that the optimal mass ratio of DOTAP to miR-223 was 1 : 0.75 for the following experiments. Then the other lipid materials (PI, DSPE-PEG2k, Chol, and Tween-80) covered outside were added into the organic phase containing DOTAP-miR-223. Following rotary evaporation, the EGCG solution was added and the liposomes were formed.

The liposomes were extruded through a polycarbonate film to form nanoparticles with a uniform size. The nanoparticle size of lip@EGCG/miR-223 was 91.28 ± 2.28 nm, and the zeta potential was −36.21 ± 1.82 mV as measured by DLS (Fig. 2B). The results suggested that the liposomes prepared in this way had a smaller size, making it easier to target AS lesions which have a smaller vascular endothelial space.^28^ Also, the liposomes prepared were negatively charged as shown by the results, which ensured they had a high stability and low cytotoxicity both *in vitro* and *in vivo*. The morphological characteristics were observed by TEM (Fig. 2C), displaying that lip@EGCG/miR-223 was spherical in shape and evenly distributed in size.

The miR-223 protection effect of lip@EGCG/miR-223 was investigated by agarose gel retardation assay. As shown in Fig. 2D, no bands could be observed when naked miR-223 and RNase A were co-incubated for 5 min, which suggested that the miR-223 was easily degraded by RNase A when it was not encapsulated in the liposomes. The miR-223 band of lip@EGCG/miR-223 was still bright when incubated with RNase A for 6 h, and the result demonstrated that lip@EGCG/miR-223 could effectively protect miR-223 from the degradation by RNase A, which should make it possible for miR-223 to target tissues or cells precisely *in vivo*.

In addition, the encapsulation efficiency (EE) of lip@EGCG/miR-223 for EGCG was calculated to be 81.1% (1.622 mg mL^−1^), and for miR-223 was measured to be 98.8% (0.033 mg mL^−1^). These results suggested that EGCG and miR-223 were successfully encapsulated into the liposomes with a high encapsulation efficiency.

### 3.2. Antioxidant and anti-inflammatory effects of lip@EGCG/miR-223 *in vitro*

**Fig. 3:**
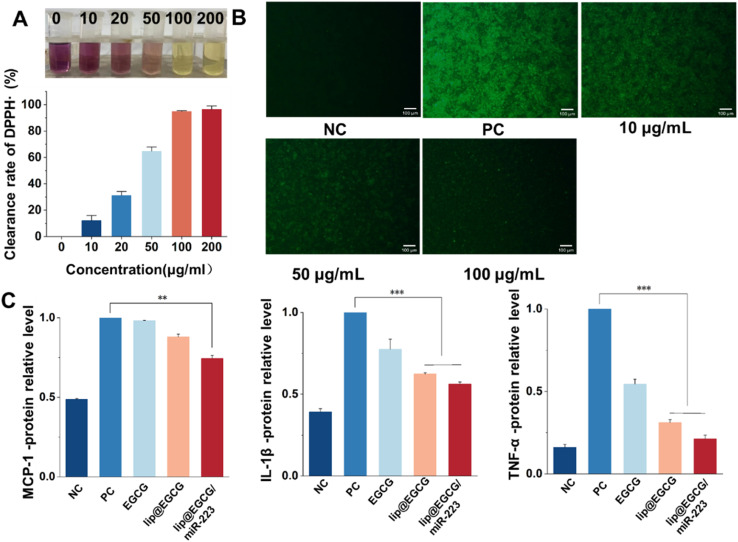
Antioxidant and anti-inflammatory effects *in vitro*. (A) DPPH˙ scavenging ability of lip@EGCG/miR-223 containing different concentrations of EGCG. (B) Fluorescence images showing the production of intracellular ROS after treatment with lip@EGCG/miR-223 containing different concentrations of EGCG and stimulated with LPS. The scale bar is 100 μm. (C) Levels of the typical inflammatory cytokines (MCP-1, IL-1β, TNF-α) detected by ELISA. *p < 0.5, **p < 0.01, ***p < 0.001, *n* = 3.

The overproduction of ROS leads to oxidative stress, which can induce tissue and cell injury that further initiates an inflammatory cycle and results in the amplification of oxidative stress.^29^ Therefore, we investigated the antioxidant effects of lip@EGCG/miR-223 at the solution level and the cellular level, respectively. To investigate the antioxidant ability of lip@EGCG/miR-223 in solution, the DPPH˙ scavenging assay was performed. DPPH˙ is a stable, nitrogen-centered free radical with characteristic absorption at 517 nm. When the antioxidant was added into DPPH˙ methanol solution, the violet DPPH radical was reduced to stable yellow DPPH molecules.^30^ The remaining DPPH radical was measured to determine the DPPH˙ scavenging capability. As shown in Fig. 3A, the color of DPPH˙ methanol solution changed from violet to yellow as the concentration of EGCG in lip@EGCG/miR-223 increased. These results indicated that lip@EGCG/miR-223 exhibited an excellent DPPH-radical-scavenging capacity.

In addition, the generation of ROS in RAW 264.7 macrophages was detected by staining with DCFH-DA, a fluorescent probe used to detect intracellular ROS. As shown in Fig. 3B, the fluorescence intensity in the cells treated with LPS (positive control) was remarkably enhanced when compared with the cells without treatment (negative control), which suggested the successful construction of the oxidation stress model. The fluorescence intensity in the cells treated with lip@EGCG/miR-223 gradually decreased as the concentration of EGCG increased. When the EGCG concentration increased to 100 μg mL^−1^, the intensity of fluorescence was similar to that of the negative control group, which suggested lip@EGCG/miR-223 effectively reduced the generation of ROS in macrophages. These results above demonstrated that lip@EGCG/miR-223 had excellent antioxidant capacity *in vitro*.

Proinflammatory cytokines (such as TNF-α, IL-1β, and MCP-1) produced by immune cells induce the formation of atherosclerotic plaques.^31^ In order to investigate the anti-inflammatory properties of lip@EGCG/miR-223 *in vitro*, the levels of typical inflammatory cytokines were measured by ELISA, and the results are displayed in Fig. 3C. Compared with the negative control group, the levels of inflammatory factors in the positive control group were significantly increased, indicating that the inflammatory model was successfully constructed. Whereas, when treated with lip@EGCG or lip@EGCG/miR-223, the amount of inflammatory factors decreased significantly. Also, the relative levels of IL-1β, MCP-1, and TNF-α for lip@EGCG were 0.624, 0.880, and 0.312 as compared with the positive control, which was also relatively lower than that of the EGCG-treated groups. These results suggested that the anti-inflammatory effect was improved when EGCG was encapsulated in liposomes, which may contribute to an enhanced uptake in RAW 264.7 cells. The inflammatory factor levels for lip@EGCG/miR-223 were 0.562, 0.744, and 0.212, respectively, suggesting that the co-loading with miR-223 had a better therapeutic effect on AS. All of the above results demonstrated that lip@EGCG/miR-223 had excellent anti-inflammatory effects *in vitro*, and the effect was improved when EGCG was loaded in liposomes.

### 3.3. Investigation of the cellular uptake behavior, expression of miR-223, and lipid-efflux promotion *in vitro*

**Fig. 4:**
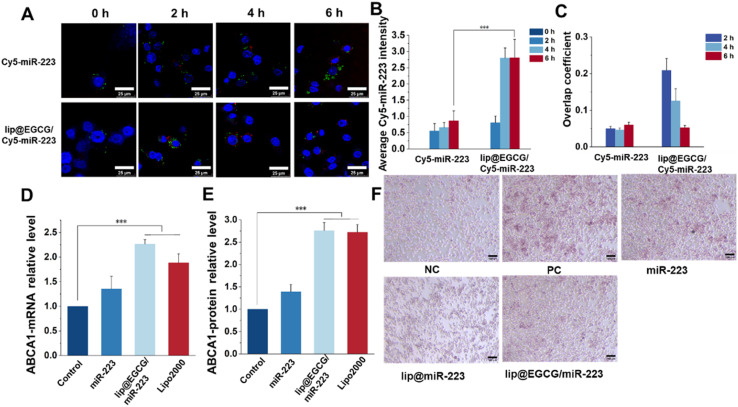
Cellular uptake, gene-delivery level, and lipid-efflux promotion *in vitro*. (A) Cellular uptake of free-Cy5-miR-223 and lip@EGCG/Cy5-miR-223 for 2, 4, and 6 h. Endosomes were stained using LysoTracker Green. The nucleus was stained with Hoechst 33 342 (blue). The scale bar is 25 μm. (B) Analysis of the Cy5 intensity according to (A) using ImageJ. (C) Overlap coefficient analysis according to (A) using ImageJ. (D) Determination of the ABCA1 gene expression by RT-qPCR. (E) Determination of the ABCA1 protein level by ELISA. (F) ORO-stained images in RAW 264.7 cells. The scale bar is 100 μm. *p < 0.5, **p < 0.01, ***p < 0.001, *n* = 3.

As reported, a low cellular uptake and lysosomal degradation are the main obstacles for the application of nanocarriers in gene drug delivery.^32^ So, the intracellular uptake and lysosomal escape capacity of lip@EGCG/miR-223 were investigated here in RAW 264.7 cells by CLSM. Cy5-labeled miR-223 was applied to trace the position of miRNA in RAW 264.7 cells, and the nuclei and lysosomes were stained with Hoechst 33 343 and LysoTracker Green, respectively. As shown in Fig. 4A, the red fluorescence of Cy5 was increased both in the free Cy5-miR-223- and lip@ECGC/Cy5-miR-223-treated groups with the increase in incubation time. However, at 4 and 6 h, the intensity of red fluorescence in the lip@EGCG/Cy5-miR-223 group was significantly higher than that in the free Cy5-miR-223 group (Fig. 4B), as analyzed by ImageJ software. The overlap coefficient of miR-223 and the lysosomes was also analyzed by ImageJ. As shown in Fig. 4C, for the free Cy5-miR-223-treated group, the overlap coefficient increased with the extension in experiment time, suggesting that the miRNAs were trapped in the lysosomes. The overlap coefficient in the lip@EGCG/Cy5-miR-223 group was relatively high at 2 h, but decreased at 4 and 6 h. This result indicated that lip@EGCG@Cy5-miR-223 was taken up by the lysosomal pathway, and could escape from the lysosomes rapidly, which may contribute to the cationic lipid material DOTAP. All of the results above suggest that lip@EGCG/miR-223 was an excellent carrier for miRNA delivery, which could not only deliver miRNAs into cells effectively, but also promote the escape of miRNAs from lysosomes to perform a gene silencing effect in the cytoplasm.

The expression of miR-223 target genes regulated by lip@EGCG/miR-223 was evaluated at both mRNA levels and protein levels. First, the upregulation of ABCA1 mRNA treated with different drugs was determined by RT-qPCR analysis. As shown in Fig. 4D, the relative level of ABCA1 mRNA was 1.35 in the free miR-223 group compared with the negative control group, and it was significantly increased to 2.27 for the lip@EGCG/miR-223-treated ones, which had a similar effect as the positive control (Lipo/miR-223).

Also, the upregulation of the ABCA1 protein treated with different drugs was determined by ELISA analysis. As shown in Fig. 4E, the relative level of ABCA1 mRNA was 1.39 in the free miR-223 group compared with the negative control group, and it was significantly increased to 2.76 for the lip@EGCG/miR-223-treated ones, which had a similar effect as the positive control (Lipo/miR-223). All of these results demonstrated that lip@EGCG/miR-223 could upregulate the expression of ABCA1, which is an important transporter for lipid efflux.^33^

Macrophages phagocytose could uptake large amounts of ox-LDL to form foam cells and thus lead to an abnormal accumulation of lipids, which is an essential hallmark of atherosclerotic lesions.^34^ Also, eliminating lipids in foam cells has been confirmed to be of great significance for atherosclerosis treatment. In this section, we thus examined the ability of lip@EGCG/miR-223 to prevent foam cell formation and lipid accumulation. As shown in Fig. 4F, considerable lipids were observed in cells incubated with ox-LDL, which was treated as the positive control group. The results indicated that a large number of foam cells were formed after ox-LDL induction. The free miR-223 group showed a limited ability to inhibit foam cell formation, whereas the lip@miR-223 group displayed a significant reduction in foam cells. Also, lip@EGCG/miR-223 had a similar foam cell formation inhibiting effect as lip@miR-223, suggesting that the co-delivery of EGCG and miR-223 had no influence on the treatment effect of miR-223. Collectively, lip@EGCG/miR-223 could promote lipid efflux by inhibiting the formation of foam cells due to the gene therapy effect of miR-223.

### 3.4. Targeting and pharmacodynamic investigations of lip@EGCG/miR-223 *in vivo*

**Fig. 5:**
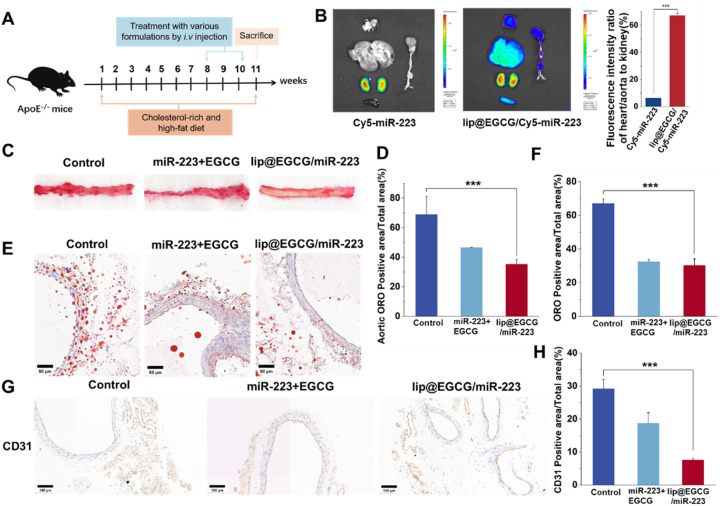
Pharmacodynamic evaluation *in vivo*. (A) Establishment of the atherosclerosis model in ApoE^−/−^ mice. (B) Fluorescence images of liposome accumulation in various organs 8 h post-injection and the relative ratio of fluorescence in the heart/aorta to the kidney. (C) ORO-stained images of aortic sections. (D) Quantitation of lesion regions (oil-red area) in the aorta tissue. (E) ORO-stained images of aortic sections. The scale bar is 50 μm. (F) and (G) Images of aortic sections stained with antibody to CD31. The scale bar is 100 μm. (H) Quantitative analysis of the CD31 positive areas relative to the total arterial wall area using ImageJ. *p < 0.5, **p < 0.01, ***p < 0.001, *n* = 3.

As atherosclerosis progresses, the connection between endothelial cells is destroyed, and neoangiogenesis occurs, making them leaky and fragile, and resulting in an EPR effect in AS lesions.^35,36^ Therefore, lip@EGCG/miR-223 could be passively targeted at AS lesions. In this section, the targeting and tissue distribution behavior of lip@EGCG/miR-223 was investigated by the AS mice model. As illustrated in Fig. 5B, for the free Cy5-miR-223-treated group, the fluorescence was mainly distributed in the kidney, and could not observed in other organs. This suggested that the free miR-223 was excreted by the kidney, and could not be delivered to the AS lesions to exert a treatment effect. However, for the lip@EGCG/miR-223-treated group, strong fluorescence intensities were even observed in the kidney of the AS model mice, while there was still an obvious fluorescence distribution in the heart and aorta area. The semiquantitative results showed that the fluorescence intensity of the heart and aorta to that of the kidney was 6.27% in the free Cy5-miR-223-treated group, while the ratio was 66.10% in the lip@EGCG/miR-223-treated group. The results displayed that the distribution of miR-223 in the heart and aorta was significantly increased when it was encapsulated in lip@EGCG/miR-223, making it a promising targeted agent for atherosclerosis therapy.

Based on the promising results above, the treatment effects of lip@EGCG/miR-223 for AS were examined by Oil red O (ORO) staining test to reveal the formation of atherosclerotic lipid plaque in the aorta. The AS model mice were administrated with glucose solution, free EGCG, and miR-223, or lip@EGCG/miR-223. Also, the aortas and aortic sections were stained with ORO. As shown in Fig. 5C, the glucose solution group showed high ORO-stained areas, suggesting distinct lipid plaques were formed by the high fat feeding. By contrast, the plaque area was slightly decreased in the free EGCG and miR-223 groups, and the plaque area was significantly decreased in the lip@EGCG/miR-223 group. In addition, consistent with this result (Fig. 5E), compared with the glucose solution group and free EGCG and miR-223 groups, observation of the ORO-stained aortic cryosections also revealed the lowest ORO-stained areas in the lip@EGCG/miR-223 group. These results revealed that lip@EGCG/miR-223 had a strong inhibitory effect on the progression of atherosclerotic plaques by promoting lipid efflux.

The platelet endothelial cell adhesion molecule-1 (CD31) is a molecule expressed in hematopoietic and immune cells and endothelial cells, and is involved in angiogenesis, platelet aggregation, and thrombosis, which are closely related to the occurrence of AS.^37^ So antibodies to CD31 were applied here to detect the development of neovascularization and to observe the morphology of the aortic vessels in the aorta. As shown in Fig. 5G, in the glucose-solution-treated group as well as free EGCG and miR-223 treated groups, the CD31 staining level was significantly higher than that in the lip@EGCG/miR-223-treated group. Also, compared with the lip@EGCG/miR-223-treated group, much more micro-angiogenesis was observed in the glucose-solution-treated group. These results proved that lip@EGGC/miR-223 could promote the normalization of vessels by inhibiting neovascularization.

### 3.5. Mechanisms of lip@EGCG/miR-223 in the treatment of AS *in vivo*

**Fig. 6:**
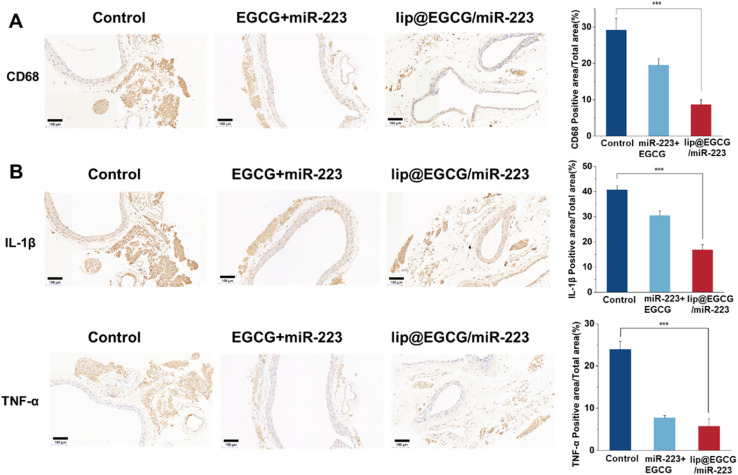
Histochemistry analyses of aortic sections after different treatments. The scale bar is 100 μm. (A) Images of aortic sections stained with antibody to CD68 and quantitative analysis of the (CD68) positive areas relative to the total arterial wall area using ImageJ. (B) Images of aortic sections stained with an antibody to IL-1β, and antibody to TNF-α, and quantitative analysis of the (IL-1β, TNF-α) positive areas relative to the total arterial wall area using ImageJ. *p < 0.5, **p < 0.01, ***p < 0.001, *n* = 3.

In order to reveal the mechanisms responsible for the *in vivo* anti-atherosclerotic activity of lip@EGCG/miR-223, histology and immunohistochemistry tests were performed. CD68 was the most reliable marker for macrophages, so anti-CD68 antibodies were applied to detect the accumulation of macrophages in aortic areas.^38^ As shown in Fig. 6A, compared with the glucose-solution-treated group as well as free EGCG- and miR-223-treated groups, the group treated with lip@EGCG/miR-223 displayed the lowest level of foam cells in aortic vessels, which were macrophages full of lipid material. This indicated that lip@EGCG/miR-223 could effectively reduce the accumulation of macrophages in aortic areas, which plays a critical role in the development of AS.

In addition, we investigated the anti-inflammatory effect of lip@EGCG/miR-223 *in vivo* by histochemistry analysis of two typical inflammatory cytokines (IL-1β and TNF-α) in the aorta. As shown in Fig. 6B, the lowest expressions of IL-1β and TNF-α were observed in the lip@EGCG/miR-223 group, which suggested that lip@EGCG/miR-223 exhibited excellent anti-inflammatory activity. Since atherosclerosis has been widely reported as a chronic inflammatory disease,^39^ the outstanding anti-inflammatory effect of lip@EGCG/miR-223 makes it a promising agent for AS treatment.

Together, these results indicated that when administrated with lip@EGCG/miR-223, the inflammatory environment in aortic areas of the AS model mice was relieved, which contributed to the excellent antioxidant and anti-inflammatory ability of EGCG. The antioxidant effect of EGCG could also decrease the generation of oxidized low-density lipoprotein (ox-LDL) to inhibit the formation of foam cells in aortic areas. Moreover, miR-223 encapsulated in lip@EGCG/miR-223 could promote lipid efflux to further eliminate foam cells in aortic areas.

### 3.6. Biosafety evaluation of lip@EGCG/miR-223

**Fig. 7:**
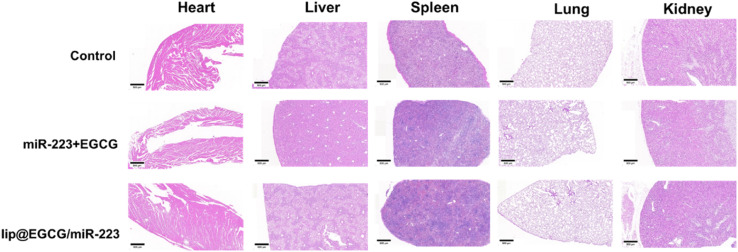
H&E staining of the major organs after the last injection treatment. The scale bar is 500 μm.

The possible side effect of lip@EGCG/miR-223 was investigated by H&E staining of histological sections of the main organs (heart, liver, spleen, lung, and kidney). As shown in Fig. 7, no distinguishable pathological features were observed in sections of each group, which indicated that lip@EGCG/miR-223 did not harm the main organs of the mice.

## 4. Conclusion

In summary, we developed a novel lipid nano-drug delivery system, co-encapsulating the antioxidant and anti-inflammatory drug EGCG as well as lipid-efflux-promoting gene miR-223, for the effective treatment of atherosclerosis in this study. By an improved thin film hydration method, both EGCG and miR-223 could be loaded into liposomes with a high encapsulation efficiency. Moreover, compared with the cationic liposomes widely applied for gene delivery, gene drugs were encapsulated into the core rather than absorbed on the surface of the liposomes, which provided the liposomes with an excellent protective effect for genes. The cellular experiments indicated that lip@EGCG/miR-223 was efficiently endocytosed in macrophages by a lysosomal pathway, and the drugs in liposomes could rapidly escape from lysosomes to exert their treatment effect. As an excellent antioxidant and anti-inflammatory agent, EGCG encapsulated in lip@EGCG/miR-223 showed a brilliant effect both in solution and at the cellular levels. Also, miR-223 encapsulated in lip@EGCG/miR-223 could effectively upregulate the expressions of ABCA1 mRNAs and proteins, to promote lipid efflux from macrophages. By the two mechanisms mentioned above, the formation of foam cells, which is an essential hallmark of atherosclerotic lesions, was significantly inhibited when treated with lip@EGCG/miR-223. After *i.v.* administration in AS model mice, lip@EGCG/miR-223 could be accumulated in atherosclerotic plaques *in vivo* by passive targeting, to effectively eliminate lipid accumulation in the aorta. Also, the preliminary mechanism research revealed that the arterial vessels in AS were normalized by the anti-inflammatory and lipid-efflux-promoting effects of lip@EGCG/miR-223. Furthermore, organic toxicity experiments *in vivo* proved that lip@EGCG/miR-223 was safe as a drug-delivery system. Consequently, lip@EGCG/miR-223 is a promising therapeutic strategy for the treatment of atherosclerosis with remarkable drug loading and protective effects, as well as a targeted drug-delivery ability to treat AS. Also, for the first time we proposed the combination of antioxidant, anti-inflammatory and lipid-promoting efflux therapy for the treatment of AS, which provides a new insight into improved atherosclerosis treatment.

## Author contributions

All authors made a significant contribution to the work reported, whether that is in the conception, study design, execution, acquisition of data, analysis and interpretation, or in all these areas; took part in drafting, revising or critically reviewing the article; gave final approval of the version to be published; have agreed on the journal to which the article has been submitted; and agree to be accountable for all aspects of the work.

## Conflicts of interest

The authors declare no conflicts of interest.

## Supplementary Material

NA-006-D3NA00369H-s001
