# Supplementary figures and images for: Multifunctional liposomes Co-encapsulating epigallocatechin-3-gallate (EGCG) and miRNA for atherosclerosis lesion elimination
Source: Nanoscale Adv. 2023 Oct 30;6(1):221–32. doi: 10.1039/d3na00369h (PMC10729916; doi:10.1039/d3na00369h)

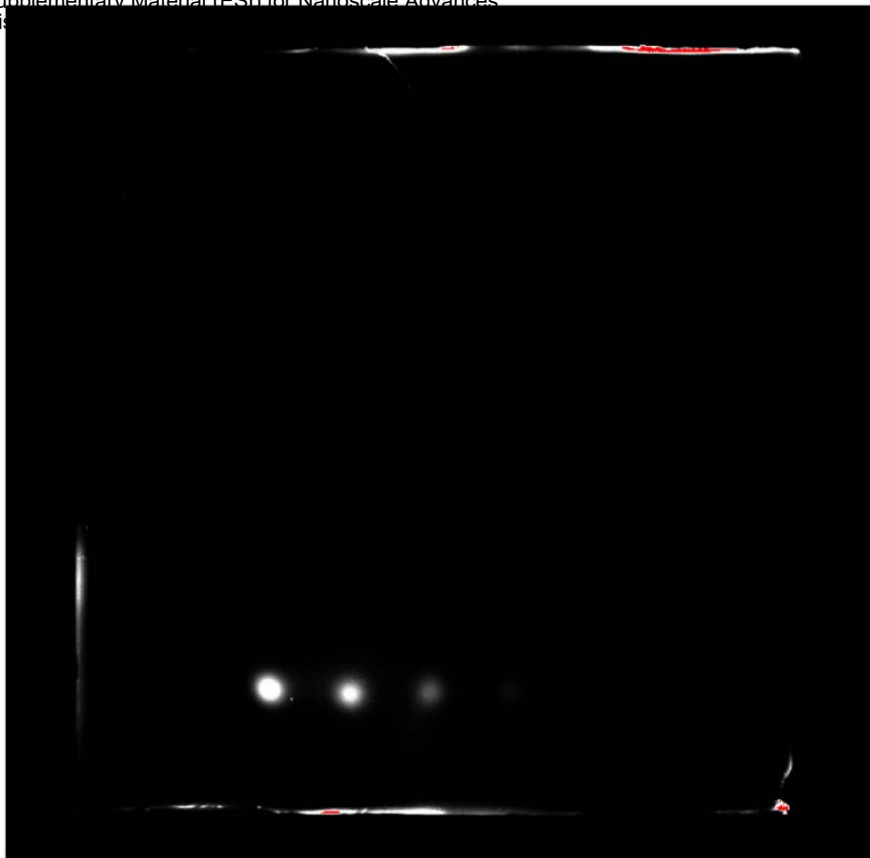

**DOTAP:miR-223 1:1.5 1:1.25 1:1 1:0.75 1:1.5**  
**(weight ratio)**

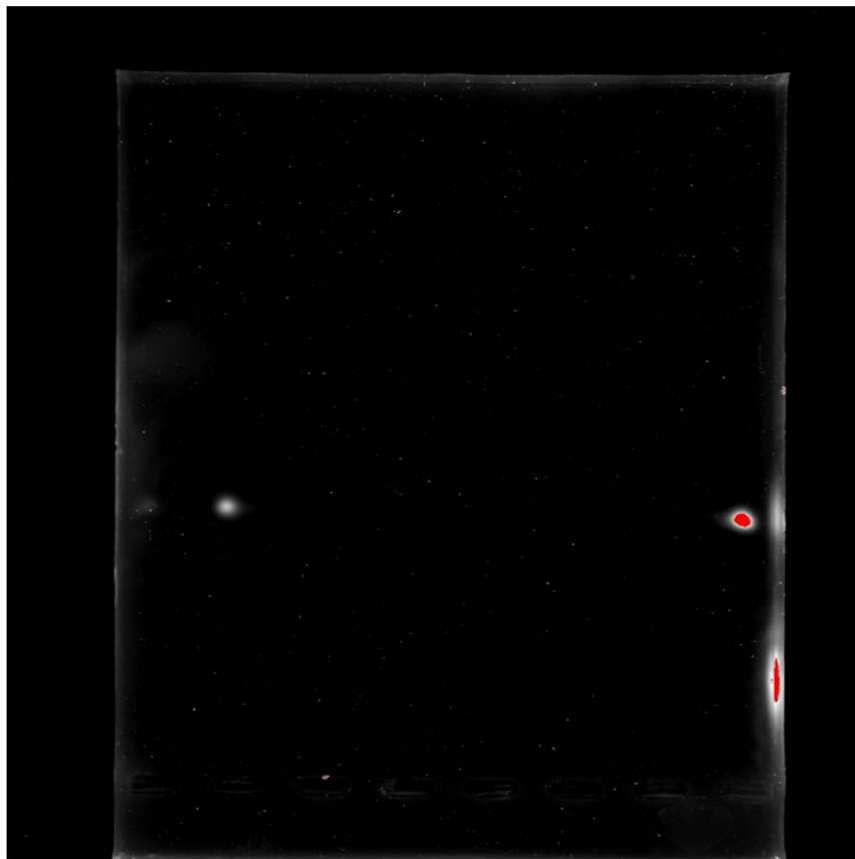

Incubation(min) 0 5 15 30 45 60 0

**Naked miR-223**

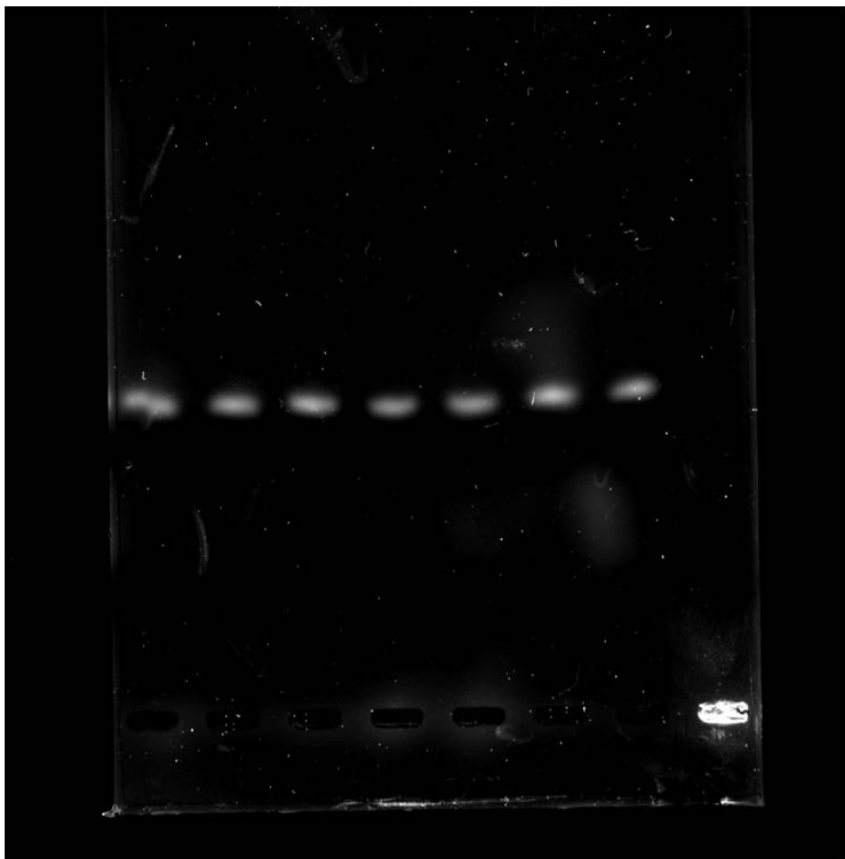

Incubation(h) 0 0.5 1 1.5 2 4 6

lip@EGCG/miR-223

Supplement: NA-006-D3NA00369H-s001 [file NA-006-D3NA00369H-s001.pdf]
